# Multi-scale triglyceride crystal network analysis using a benchtop ultra-small-angle X-ray scattering instrument

**DOI:** 10.1039/d6ra00082g

**Published:** 2026-03-05

**Authors:** Kenneth Q. K. Truong, Alejandro G. Marangoni

**Affiliations:** a Department of Food Science, University of Guelph Guelph Ontario N1G2W1 Canada amarango@uoguelph.ca

## Abstract

Benchtop ultra-small-angle X-ray scattering (USAXS) offers a practical route to probing micron-scale structural features in soft-matter systems, provided that instrumental limitations are explicitly defined and respected. In this work, a Rigaku NANOPIX mini USAXS instrument is used to characterize hierarchical fat crystal networks, with emphasis on establishing a reliable analysis window and appropriate treatment of slit-geometry effects. Analyzer crystal rocking curves are employed to define a lower bound for quantitative analysis (*q*_min_ ≈ 3.4 × 10^−4^ Å^−1^), while counting-statistics considerations define an upper bound (*q*_max_ ≈ 1.4 × 10^−2^ Å^−1^). Data outside this window are shown to be strongly influenced by direct-beam and noise artifacts and are therefore excluded from interpretation. Within the valid *q*-range, slit-smearing effects inherent to Bonse–Hart geometries are addressed by smearing structural models using open-source SASView software rather than numerically desmearing experimental data. Using cocoa butter, commercial chocolate, and a reference triglyceride mixture as representative case studies, power-law scattering regimes are extracted and compared with synchrotron SAXS measurements over overlapping *q*-ranges. While absolute slope values vary between instruments and samples, benchtop USAXS captures consistent scattering trends, including stable power-law behavior in tempered systems and transient curvature in untempered samples that diminishes upon storage. These results demonstrate that benchtop USAXS, when interpreted within a rigorously defined *q*-window and with appropriate resolution treatment, provides a reproducible and accessible tool for comparative analysis of hierarchical fat systems. More broadly, this study outlines best practices and interpretive boundaries for laboratory-scale USAXS measurements in soft-matter research.

## Abstract 2

Benchtop ultra-small-angle X-ray scattering (USAXS) offers a practical route to probing micron-scale structural features in soft-matter systems, provided that instrumental limitations are explicitly defined and respected.

## Graphical abstract

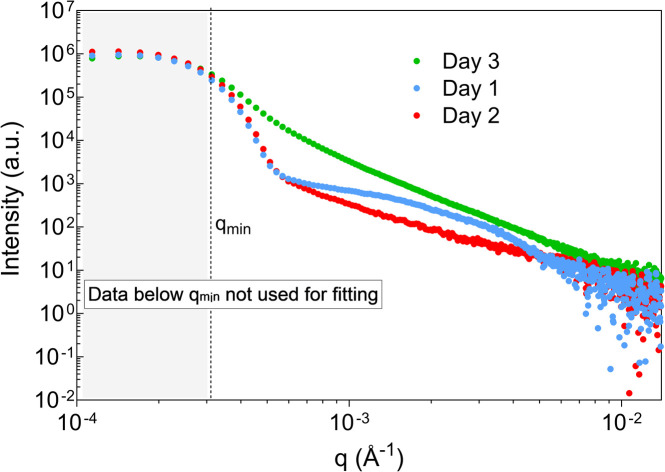

## 1 Introduction

X-ray scattering and diffraction techniques provide non-destructive access to the structural organization of crystalline, biological, and soft-matter systems across multiple length scales.^1–4^ Many functional materials, including edible fats, exhibit hierarchical organization in which nanoscale crystalline order, mesoscale aggregation, and microscale network formation coexist and collectively influence macroscopic behavior.^5–8^ As a result, scattering techniques spanning wide-angle X-ray scattering (WAXS), small-angle X-ray scattering (SAXS), and ultra-small-angle X-ray scattering (USAXS) are routinely combined to probe structure across these regimes.

In the WAXS region (*q* ≈ 1–5 Å^−1^), information on crystalline polymorphism and lattice spacings is obtained, while the SAXS region (*q* ≈ 0.1–1 Å^−1^) provides access to nanoscale periodicities such as lamellar spacings and ordered supramolecular features. At even smaller angles, the USAXS region (*q* ≈ 10^−4^–10^−1^ Å^−1^) probes micron-scale structural features, including aggregate size, packing, and fractal dimensionality. Access to this regime is essential for characterizing hierarchical networks formed by aggregated crystalline nanoplatelets in edible fat systems.^3^

Despite its importance, USAXS measurements are most commonly performed at synchrotron facilities, where beam brilliance, collimation, and optical flexibility enable access to very small scattering angles.^9^ In edible fat research, the majority of reported USAXS studies have therefore relied on synchrotron beamlines.^3^ While these facilities provide unparalleled capabilities, access is limited by competitive proposal-based allocation, infrequent scheduling cycles, travel costs, and logistical constraints.^10^ As a result, there is growing interest in laboratory-scale alternatives that can provide routine access to the ultra-small-angle regime, particularly for pre-characterization, exploratory studies, and longitudinal measurements.

Recent advances in laboratory instrumentation have made benchtop USAXS systems increasingly accessible. These instruments typically employ Bonse–Hart crystal optics and slit geometries to achieve high angular resolution, allowing extension into the ultra-small-angle regime under laboratory conditions.^11–13^ However, the use of slit geometry introduces instrument-specific resolution effects, including slit smearing and direct-beam artifacts at very low *q*, which complicate data interpretation. Without careful definition of the usable *q*-range and appropriate treatment of resolution effects, there is a risk of overinterpreting features that are dominated by instrumental rather than structural contributions.

Accordingly, the objective of the present work is not to provide a comprehensive crystallization or polymorphism study of edible fats, but rather to establish best practices for the responsible use and interpretation of benchtop USAXS data. Using a Rigaku NANOPIX mini instrument as a representative example, we demonstrate how analyzer crystal rocking curves can be used to define a reliable lower bound (*q*_min_) for quantitative analysis, and how practical counting-statistics considerations define an upper bound (*q*_max_). Within this explicitly defined window, we show how widely available open-source software (SASView) can be used to account for slit-smearing effects through model convolution rather than numerical desmearing of experimental data.

Edible fat systems, including cocoa butter, commercial chocolate, and a reference triglyceride mixture, are used as illustrative case studies because their hierarchical organization spans the length scales accessible to USAXS. Comparisons with synchrotron SAXS data collected over overlapping *q*-ranges are used to assess the extent to which benchtop measurements reproduce structural trends, while also highlighting expected limitations arising from instrumental constraints. By emphasizing interpretive boundaries alongside practical capabilities, this work aims to provide a clear framework for the routine and reproducible application of benchtop USAXS in soft-matter and food-materials research.

## 2 Experimental section

### 2.1 Materials and sample preparation

Cocoa butter (CB) was purchased from Raw Elements (Rockwood, ON, Canada). Lindt Excellence Cocoa 99% chocolate (Chocoladefabriken Lindt & Sprüngli AG, Kilchberg, Switzerland) was purchased from a local market (United Kingdom). Glass capillary tubes (1.56 × 2.00 × 50 mm) were acquired from Vitrex Medical A/S (Herlev, Denmark).

Unrefined CB was neutralized to remove free fatty acids. The CB was heated to 60 °C, then a 15% sodium hydroxide treatment was added based on the percentage of free fatty acid determined. The mixture was stirred for 20 minutes at room temperature, followed by an additional 5 minute mixing step at 70 °C. The sample was centrifuged to separate the soap layer, then washed with hot water in a separatory funnel. The neutralized CB was heated to 110 °C then bleached while stirring using 0.5% bentonite bleaching clay for further refinement. After 25 minutes, the sample was filtered using a Whatman Grade 4240 mm filter paper.

Refined, tempered CB was produced as follows. The CB was melted at 60 °C to remove any crystal history. The CB was left to cool at room temperature, down to ∼34 °C and monitored using a thermocouple. Using a tempering table affixed to a cooler set to 20 °C, two-thirds of the CB was transferred to the table and sheared using scrapers until the viscosity increased. Then, the tabled CB was transferred back into the beaker containing the remaining one-third CB, mixed, and the temperature checked. If the temperature was >28 °C, the shearing/transferring steps were repeated until the temperature read ∼27–28 °C. The tempered CB was poured into 35.0 × 19.0 × 5.0 mm plastic molds and allowed to set at 5 °C for an hour before transferring to an incubator at 18 °C to finish crystallizing. After ∼24 hours, the tempered samples were removed from the molds and stored at 18 °C until analysis.

Refined, tempered CB and 99% Lindt dark chocolate were chopped using a razor knife into small particles, enough to load into glass capillary tubes with sufficient packing. The untempered (UT) CB sample was loaded by melting refined CB and transferring into a capillary using a syringe and allowed to statically crystallize at ∼18 °C until analysis.

### 2.2 Rigaku NANOPIX mini description

Laboratory ultra-small-angle X-ray scattering (USAXS) experiments were performed using a Rigaku NANOPIX mini system. The instrument is based on a Bonse–Hart crystal-optics configuration, in which channel-cut crystals are used for both beam collimation and angular analysis.^11^ This geometry enables access to very small scattering angles under laboratory conditions, with resolution characteristics that differ from those of pinhole-collimated SAXS instruments.

A sealed Cu Kα X-ray tube (*λ* = 1.54 Å) was used as the radiation source. Beam conditioning was achieved using Rigaku's Cross Beam Optics (CBO) unit, which employs graded multilayer mirrors to select Cu Kα radiation and produce a quasi-parallel incident beam.^12^ A Soller slit with a total acceptance angle of 5° (±2.5° relative to the beam axis) was mounted upstream of a two-bounce Ge(220) channel-cut monochromator crystal to restrict beam divergence and suppress background scattering.^13^

On the detector side, scattered X-rays were analyzed using a four-bounce Ge(220) channel-cut crystal prior to detection with a D/teX Ultra 250 one-dimensional silicon strip detector comprising 256 channels. Beam intensity was regulated using an attenuator box positioned upstream of the sample; no detector-side attenuator was employed. Samples were mounted on a motorized stage within the enclosed instrument chamber using standard capillary holders. Measurements were performed under static sample conditions; no *in situ* mechanical deformation or flow was applied.

### 2.3 Resolution and baseline analysis

The usable scattering-vector range for quantitative analysis was established experimentally to ensure that subsequent interpretation was restricted to regions dominated by sample scattering rather than instrumental contributions. The scattering vector was defined as
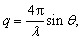
where *λ* is the X-ray wavelength and *θ* is half the scattering angle.

Analyzer crystal rocking curves of the direct beam were acquired following standard optical alignment by scanning the receiving-side analyzer crystal through the beam. Each scan produced a finite-width intensity peak reflecting the effective angular resolution of the instrument. The full width at half maximum (FWHM) of this peak was used as a practical resolution metric, with the minimum resolvable half-angle defined as
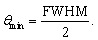


From this value, a lower bound for quantitative analysis was calculated as
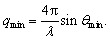


Rocking-curve measurements were repeated on multiple dates to assess instrumental stability. Across all sessions, the calculated *q*_min_ values were consistent, indicating stable instrument performance.

An upper bound for reliable analysis, *q*_max_, was determined empirically from measured scattering profiles by identifying the *q*-range beyond which counting-statistics noise dominated the signal. Above this threshold, intensity fluctuations precluded stable fitting, and data were excluded from further analysis. Together, *q*_min_ and *q*_max_ define the analysis window used throughout this study.

Background scattering was evaluated using empty glass capillaries (inner diameter 1.5 mm) measured under identical instrumental conditions. Variations in low-*q* intensity were observed between nominally identical capillaries, particularly near the direct beam. To minimize baseline bias, background subtraction was performed using capillaries exhibiting lower low-*q* intensity, with multiple replicates acquired to assess reproducibility. Background subtraction and subsequent fitting were restricted to the established analysis window.

### 2.4 Slit-smearing and data treatment

The Bonse–Hart geometry employed by the benchtop USAXS instrument introduces slit smearing due to asymmetric angular resolution along orthogonal directions. As a result, the measured intensity at a given nominal scattering vector represents an average over a finite *q*-range rather than a point measurement, leading to broadening and attenuation of sharp structural features relative to pinhole-collimated measurements.

Slit-smearing effects were treated by convolving structural models with an instrument-specific slit function prior to fitting, rather than numerically desmearing experimental data. This approach preserves experimental uncertainties and reduces the risk of noise amplification or artifact introduction.^14–19^ Model fitting was performed using the open-source SASView software package. Resolution smearing is handled *via* model-data convolution with options for slit, pinhole, and 2D smearing, and can use instrumental-derived resolution values (d*Q*) from data reduction or user-specified values. The instrument resolution was specified as slit type, with slit length = 0.178 Å^−1^ (from instrumental geometry with an angular divergence of 2.5°), and an effectively small slit width (slit length >> slit width).^20^

For power-law scattering regimes with exponent *p* > 1, slit smearing results in a systematic reduction of the apparent slope by approximately one unit relative to the corresponding pinhole-collimated measurement.^21–23^ Accordingly, reported power-law exponents correspond to pinhole-equivalent values inferred through model smearing, while measured slit-smeared curves exhibit slopes consistent with *p* − 1 behavior. All reported exponents were obtained within explicitly stated *q*-ranges confined to the validated analysis window.

### 2.5 Synchrotron small-angle X-ray scattering (SAXS)

Small-angle X-ray scattering (SAXS) experiments were performed on the I22 beamline at the Diamond Light Source (Oxfordshire, United Kingdom).^24^ The wavelength utilized was 1.24 Å (10.0 keV). A Pilatus P3-2 M detector (172 × 172 µm^2^ pixel size, 254 × 289 mm^2^ area) with a sample to detector distance of 9.753 m recorded the SAXS measurements, covering a *q*-range of ∼0.01–0.15 Å. Silver behenate was used for detector calibration and a Canberra PD-300 PIPS diode for flux calibration.^24^ One frame acquisition of 0.1 s was performed for each sample. The Data Analysis WorkbeNch (DAWN) software package was used to reduce two-dimensional detector images to one-dimensional intensity *versus q*-plots, including a data correction sequence.^25–27^ GraphPad Prism (Boston, MA, USA) was used for final data plotting.

## 3 Results and discussion

The Results section is organized to first establish the practical limits and interpretive boundaries of benchtop ultra-small-angle X-ray scattering (USAXS) measurements, before applying the validated analysis framework to representative fat systems. We begin by defining a reliable *q*-range for quantitative interpretation based on experimentally measured instrument resolution, followed by an examination of background-related limitations at very low *q* and the treatment of slit-smearing effects inherent to Bonse–Hart geometries. With these constraints explicitly established, we then apply the resulting analysis approach to cocoa butter, chocolate, and a reference triglyceride mixture, using comparisons with synchrotron SAXS data to assess consistency over overlapping *q*-ranges.

### 3.1 Defining a reliable USAXS analysis window

**Fig. 1:**
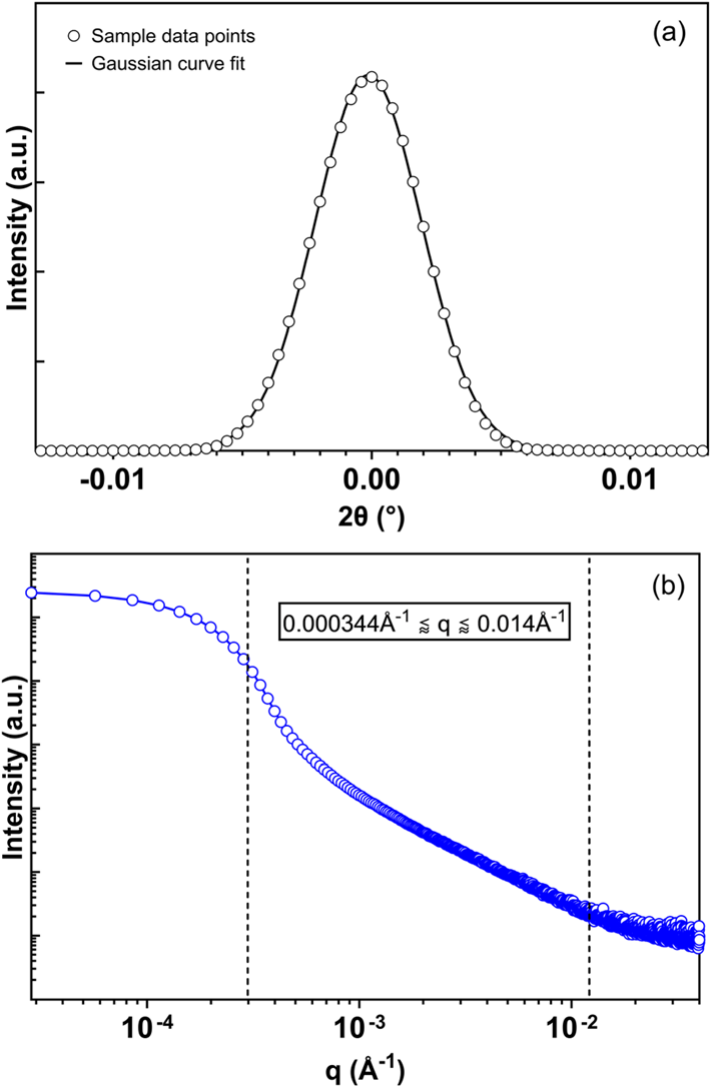
(a) Crystal rocking curve sample data fitted to a Gaussian function; (b) one-dimensional scattering pattern of a sample cocoa butter with the NANOPIX mini, displaying the usable *q*-range window (*q*_min_ = 3.4 × 10^−4^ ± 1.44 × 10^−6^ Å^−1^, *q*_max_ ≈ 1.4 × 10^−2^ Å^−1^).

The first step in the interpretation of benchtop ultra-small-angle X-ray scattering (USAXS) data is to define the range of scattering vectors over which the measured intensity is dominated by sample scattering rather than instrumental contributions. For Bonse–Hart-type geometries, this range is constrained at low *q* by the angular resolution of the analyzer crystal and at high *q* by counting-statistics limitations. Fig. 1 summarizes the experimental determination of these bounds for the benchtop USAXS instrument used in this study.

Fig. 1a shows representative analyzer crystal rocking curves acquired by scanning the receiving-side analyzer through the direct beam following standard optical alignment. The finite full width at half maximum (FWHM) of these curves reflects the effective angular resolution of the instrument and provides a practical measure of the smallest resolvable scattering angle. Using the measured FWHM values, a minimum scattering vector *q*_min_ was calculated for each measurement session. As shown in Table 1, repeated measurements acquired on different dates yielded consistent *q*_min_ values, indicating stable instrumental performance over time. Across all sessions, *q*_min_ was approximately 3.4 × 10^−4^, corresponding to an upper real-space length scale of approximately 1.8 µm. Scattering intensities measured below this threshold are therefore expected to be strongly influenced by direct-beam and resolution effects and were excluded from quantitative analysis.

**Table 1:** Theoretical minimum *q* analysis from the FWHMs of various crystal rocking curves

Date of measurement	FWHM (°)	Theoretical minimum *q* (Å^−1^)
Nov. 11, 2024	0.004849	0.0003451
Feb. 24, 2025	0.004813	0.0003343
Mar. 13, 2025	0.004820	0.0003431
May 21, 2025	0.004839	0.0003445
June 24, 2025	0.004870	0.0003466

Conversion using the scattering vector 
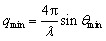
, where *λ* = 1.54 Å and 
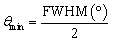
 as FWHM values for the rocking curve spectra were determined from an *I vs.* 2*θ* plot (Fig. 1a).

**Fig. 2:**
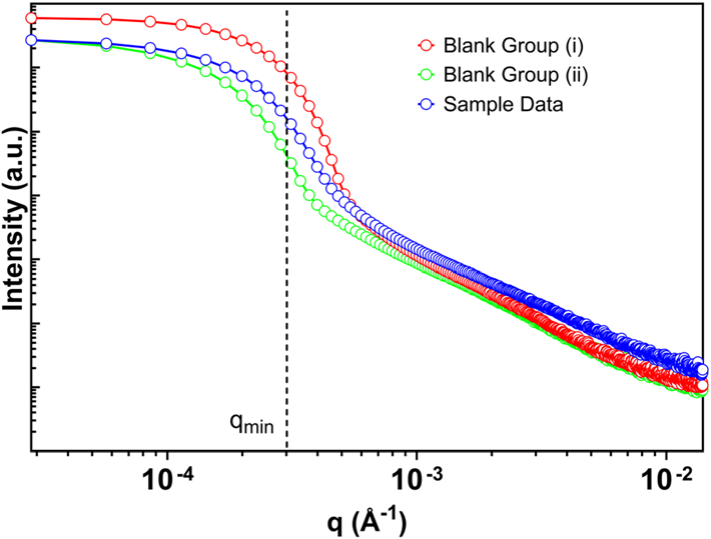
Example of raw data from empty blank capillary scattering of two groups: (i) higher scattering intensity and (ii) lower scattering intensity, against raw sample data.

The upper bound of the usable analysis window was determined empirically from measured scattering profiles. As shown in Fig. 1b, the signal-to-noise ratio decreases progressively at higher *q*, and beyond approximately *q* ≈ 1.4 × 10^−2^ Å^−1^ the measured intensity becomes dominated by counting-statistics noise. In this region, fitted parameters are not stable, and data were therefore excluded from further analysis. The resulting analysis window, bounded by *q*_min_ and *q*_max_, is explicitly indicated in Fig. 1b.

Defining this analysis window prior to structural interpretation is essential for the responsible use of benchtop USAXS data. Within this range, measured intensity trends are reproducible and suitable for comparative analysis. Outside this range, apparent features may arise from instrumental limitations rather than sample structure. All subsequent results presented in this work are therefore restricted to the experimentally established range 3.4 × 10^−4^ ≤ *q* ≤ 1.4 × 10^−2^ Å^−1^.

### 3.2 Practical background limitations at very low scattering vectors

At very small scattering vectors, USAXS measurements are particularly sensitive to background contributions arising from the direct beam, optical scattering, and sample containment. For benchtop instruments employing Bonse–Hart geometries, these effects can dominate the measured intensity below the experimentally established *q*_min_, making reliable background subtraction impractical in this region. Fig. 2 illustrates this limitation using representative measurements of empty capillaries acquired under identical instrumental conditions.

As shown in Fig. 2, empty capillaries of the same nominal dimensions exhibit measurable differences in low-*q* scattering intensity. Two classes of background behavior are observed: capillaries whose low-*q* intensity lies above that of a representative sample and capillaries whose intensity lies below it. These differences become most pronounced at *q* values approaching the direct beam, where small variations in capillary geometry, alignment, or surface quality can produce disproportionate changes in the measured signal.

This variability has direct implications for background subtraction. When background intensity approaches or exceeds sample intensity at very low *q*, subtraction can artificially modify the slope of the resulting scattering curve, leading to biased power-law exponents or spurious curvature. In such cases, apparent features may reflect the choice of background rather than sample scattering. The effect is most severe below *q*_min_, where the measured intensity is already strongly influenced by instrumental resolution and direct-beam contributions.

To minimize baseline bias, background subtraction in this study was performed using empty capillaries exhibiting lower low-*q* intensity, with multiple replicates acquired to assess reproducibility, which exhibited minimal variability in subsequently fitted parameters. However, this approach does not render data below *q*_min_ quantitatively meaningful. Rather, the results in Fig. 2 demonstrate why interpretation in this region should be avoided altogether. For this reason, all structural analyses presented here are restricted to the analysis window defined in Section 3.1, where background contributions are subordinate to sample scattering and fitted parameters are robust.

### 3.3 Treatment of slit-smearing effects using model convolution

The asymmetric angular resolution inherent to Bonse–Hart USAXS geometries results in slit smearing of the measured scattering intensity. Consequently, the reported intensity at a given nominal scattering vector represents an average over a finite *q*-range rather than a point measurement, with the magnitude of smearing increasing toward lower *q*. This effect is intrinsic to the instrument geometry and must be accounted for explicitly to enable meaningful quantitative analysis within the established analysis window.

**Fig. 3:**
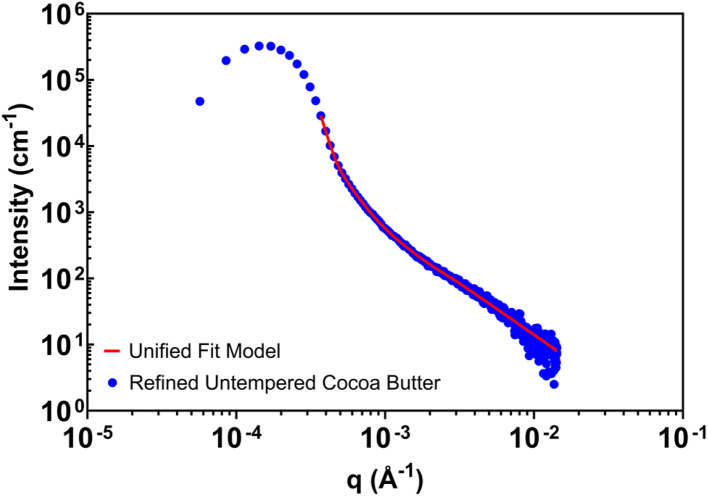
Illustrative example of a SASView fit using the Unified Fit model and refined, untempered cocoa butter.

Fig. 3 illustrates the impact of slit smearing on model fitting using a representative scattering profile analyzed with SASView. Rather than numerically desmearing experimental data, which can amplify noise and introduce artifacts, slit-smearing effects were treated by convolving structural models with an instrument-specific slit function prior to fitting. This approach preserves the experimental uncertainty structure and allows fitting to be performed directly on the measured, slit-smeared data.

For power-law scattering regimes with exponent *p* > 1, slit smearing leads to a systematic reduction in the apparent slope by approximately one unit relative to the corresponding pinhole-collimated measurement. This relationship provides a practical basis for interpreting slopes obtained from slit-smeared data within the validated *q*-range. Accordingly, reported power-law exponents correspond to pinhole-equivalent values inferred through model smearing, while the measured curves exhibit slopes consistent with *p* − 1 behavior.

Fig. 3 is presented as an illustrative example of the analysis strategy rather than as a detailed structural interpretation of a specific sample. Its purpose is to demonstrate how slit-smearing effects can be incorporated into routine analysis using widely available software, ensuring that fitted parameters reflect sample scattering rather than instrumental resolution. With slit smearing treated in this manner, we next present subsequent qualitative comparisons between benchtop USAXS and pinhole-collimated synchrotron SAXS measurements over overlapping *q*-ranges before summarizing quantitative parameters.

### 3.4 Application to tempered and untempered cocoa butter

**Fig. 4:**
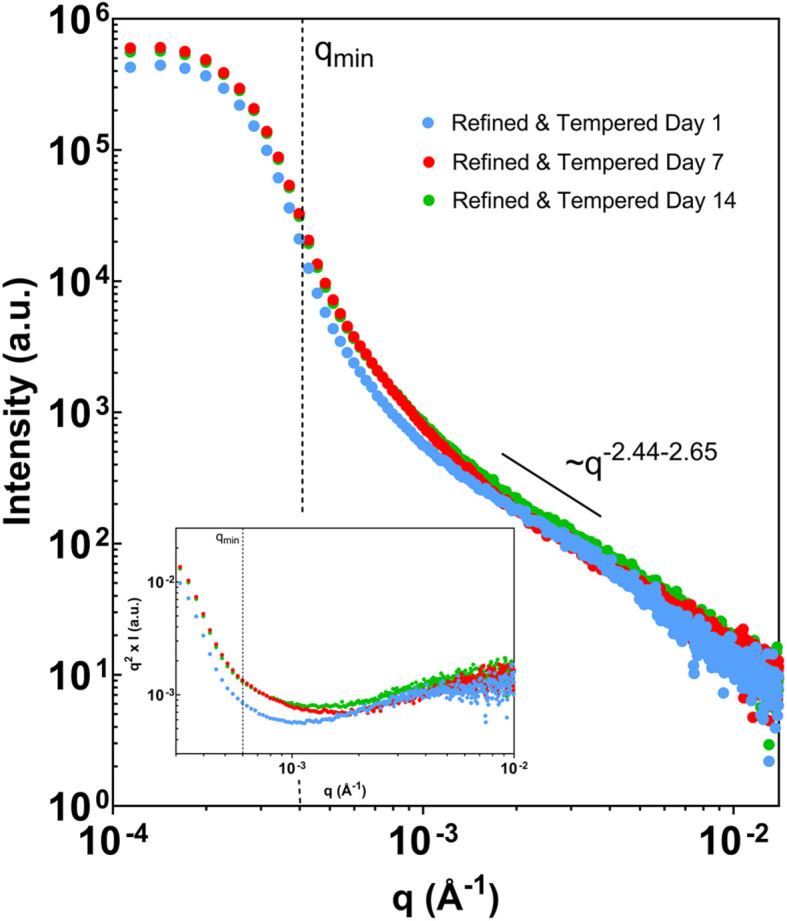
Refined, tempered cocoa butter across a two-week period at 18 °C storage. Scattering curves have been background-corrected. The inset depicts a Kratky representation of the same data (*q*^2^*I*(*q*) *vs. q*).

**Fig. 5:**
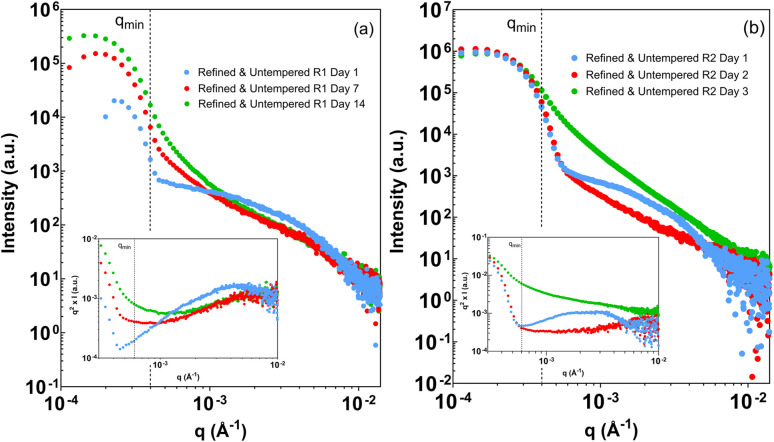
Refined, untempered cocoa butter samples across (a) a two-week period and (b) 3 day period, crystallized statically. Scattering curves have been background corrected. The inset depicts a Kratky representation of the same data (*q*^2^*I*(*q*) *vs. q*).

With the usable *q*-range and data-treatment strategy established, benchtop USAXS measurements were applied to cocoa butter systems prepared under tempered and untempered conditions. Fig. 4 and 5 present representative scattering profiles acquired within the analysis window defined in Section 3.1, with all data treated consistently for background subtraction and slit-smearing effects.

Fig. 4 shows scattering profiles for refined cocoa butter crystallized under tempered conditions and stored at 18 °C. Across multiple storage times spanning two weeks, the measured intensity exhibits a single dominant power-law regime over the accessible *q*-range. The fitted power-law exponents remain within a narrow range, indicating reproducible scattering behavior over time. No distinct Guinier regions or pronounced curvature are observed within the validated window, and the corresponding Kratky representations show no clear maxima or inflection points. Within the constraints of the measurement range, these results indicate a stable scattering regime whose dominant features do not evolve appreciably over the timescale examined.

Fig. 5 shows scattering profiles for refined cocoa butter prepared without controlled tempering. Two independently prepared samples are shown to illustrate the variability observed under these conditions. In both cases, early measurements exhibit curvature or broad bends within the measured *q*-range, visible in both the intensity profiles and the corresponding Kratky representations. With increasing storage time, these features diminish, and the scattering progressively approaches a single power-law regime similar to that observed for tempered samples. While the detailed evolution differs between replicates, the overall trend of decreasing curvature and stabilizing slope is reproducible.

Throughout this section, differences between tempered and untempered samples are reported strictly in terms of observable scattering behavior within the established analysis window. The presence or absence of curvature is interpreted as a change in the dominant length scales contributing to the measured intensity, without attributing specific aggregation or growth mechanisms. Sample-to-sample variability is treated as an intrinsic feature of the system rather than as an instrumental effect.

Taken together, Fig. 4 and 5 demonstrate that benchtop USAXS, when analyzed within a rigorously defined *q*-range, can reproducibly distinguish between stable and evolving scattering regimes in cocoa butter systems, while remaining sensitive to variability arising from sample preparation and storage history.

### 3.5 Comparison with synchrotron SAXS over overlapping *q*-ranges

**Fig. 6:**
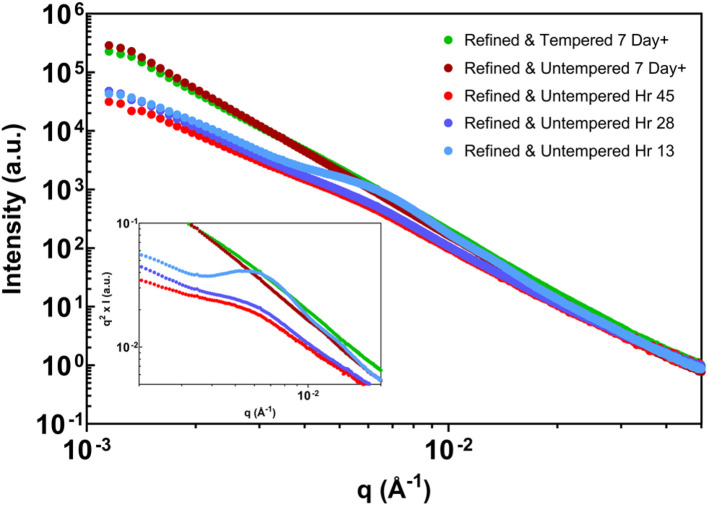
Small-angle X-ray scattering results (*I vs. q*) of refined cocoa butter collected from the I22 beamline at the Diamond Light Source synchrotron. The SAXS detector was set 9.753 m from the sample, overlapping with the USAXS region. Scattering curves have been background-corrected. The inset depicts a Kratky representation of the same data (*q*^2^*I*(*q*) *vs. q*).

To examine how benchtop USAXS measurements compare with large-scale facilities within a shared length-scale window, cocoa butter samples prepared under the same protocols were also measured using a pinhole-collimated SAXS configuration at the I22 beamline of the Diamond Light Source. Fig. 6 shows representative scattering profiles obtained at the synchrotron and reduced to one-dimensional intensity curves using standard beamline procedures. The detector distance was selected to provide overlap with the upper portion of the benchtop USAXS analysis window, enabling direct comparison of scattering behavior over a limited but common *q*-range.

Within this overlapping region, both benchtop USAXS and synchrotron SAXS measurements exhibit qualitatively similar scattering regimes. Tempered cocoa butter samples display a single dominant power-law region without pronounced curvature, whereas untempered samples show curvature or bends at early storage times that diminish with further storage. These features are observed across both instruments when comparisons are restricted to the same *q*-range, indicating that the benchtop system captures the same dominant scattering regimes accessible at the synchrotron within this shared window.

At the same time, differences in fitted power-law exponents are evident between instruments and between nominally similar samples, as summarized in Table 2. Such differences are expected given the stochastic nature of crystallization, sample-to-sample variability, and the sensitivity of fitted slopes to the position of curvature relative to the measurement window. Importantly, comparable variability is observed within measurements performed on each instrument independently and should not be interpreted as arising from instrumental inadequacy.

**Table 2:** Comparison of power-law slopes between NANOPIX mini and Diamond's I22 Beamline

Cocoa butter sample	Storage time (NANOPIX mini)	Power-law slope (NANOPIX mini)^a^	Storage time (I22)	Power-law slope (I22)
Unrefined tempered	1 Day	2.48 ± 0.06	14+ Days	2.88
	7 Days	2.43 ± 0.05		
	14 Days	2.46 ± 0.06		
Refined tempered	1 Day	2.44 ± 0.07	14+ Days	3.33
	7 Days	2.65 ± 0.06		
	14 Days	2.59 ± 0.05		
Refined untempered replicate 1	1 Day	2.13 ± 0.06	13 Hours	2.71
	7 Days	2.26 ± 0.09	28 Hours	2.89
	14 Days	2.47 ± 0.07	45 Hours	2.64
Refined untempered replicate 2	1 Day	2.94 ± 0.04	14+ Days	3.57
	2 Days	2.91 ± 0.12		
	3 Days	3.54 ± 0.02		

a Power-law slopes were fitted to a slit-smeared model representative of unsmeared data; errors represent between-blank variability within background group (ii) (Section 3.2).

The extent of the overlapping *q*-range necessarily limits the degree of quantitative comparison that can be made. With approximately one decade of overlap, the comparison is not intended to resolve detailed structural features or hierarchical organization. Rather, it serves to establish that benchtop USAXS reproduces the same qualitative scattering behavior observed at a synchrotron facility when both datasets are interpreted within equivalent q-ranges and with appropriate treatment of instrumental resolution.

Taken together, the comparison presented in Fig. 6 and Table 2 demonstrates that benchtop USAXS provides access to the same dominant scattering regimes as synchrotron SAXS over shared length scales, while underscoring the importance of restrained interpretation when comparing data acquired under different instrumental configurations.

### 3.6 Reference systems: commercial chocolate and a model triglyceride mixture

In addition to cocoa butter, two reference systems were examined to provide contextual benchmarks for the benchtop USAXS analysis: a commercial dark chocolate formulation and a model triglyceride mixture consisting of 10 wt% tristearin (SSS) in triolein (OOO). These systems were selected because their dominant scattering behavior has been reported previously and therefore provides useful reference points for evaluating the consistency of benchtop measurements within the validated analysis window.

**Fig. 7:**
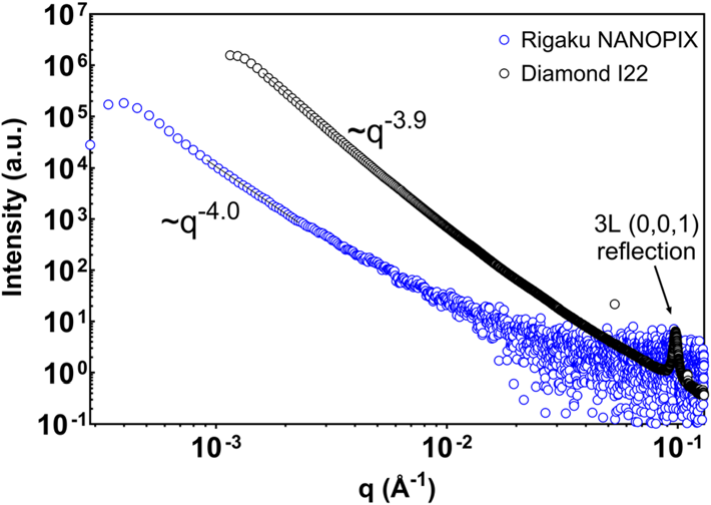
Scattering of commercial dark chocolate from NANOPIX mini (slit) and Diamond's I22 Beamline (pinhole). NANOPIX mini model has been slit-smeared, representative of a desmeared power-law.

Fig. 7 shows scattering profiles for commercial dark chocolate measured using both the benchtop USAXS instrument and synchrotron SAXS. Within the overlapping *q*-range, both measurements exhibit a single dominant power-law regime with an exponent close to four. This behavior is characteristic of Porod scattering from large dispersed solid particles, such as sugar and cocoa powder, which dominate the scattering signal in chocolate at these length scales.^28^ When slit-smearing effects are treated appropriately, the benchtop USAXS data reproduce this expected behavior within the shared *q*-range. Higher-*q* features resolved in the synchrotron measurement, including lamellar reflections, fall outside the benchtop analysis window and are therefore not interpreted here.

**Fig. 8:**
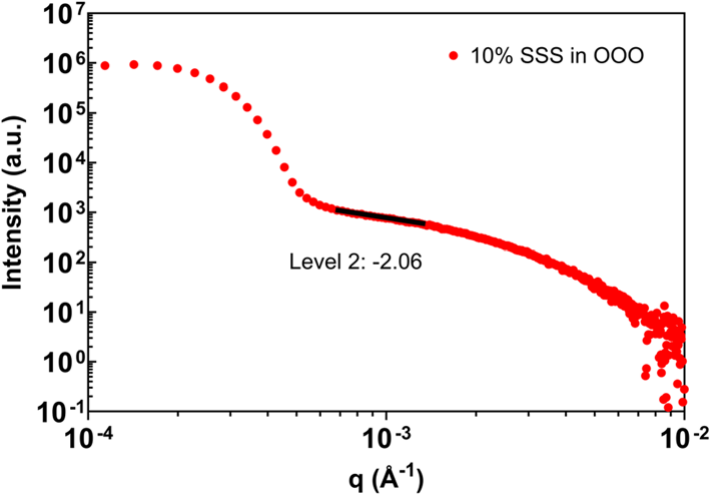
Scattering of 10% SSS in OOO measured after 12 d of storage with a level-2 slope value obtained using unified fit.

A second reference system is shown in Fig. 8, consisting of 10 wt% SSS in OOO measured after extended storage. This mixture has previously been characterized using USAXS and exhibits a level-2 power-law regime with an exponent close to two.^29^ When analyzed using the benchtop USAXS instrument within the validated *q*-range, the measured scattering reproduces the expected power-law behavior. Features reported at lower or higher *q* in synchrotron measurements are not discussed here, as they lie outside the experimentally established limits of the benchtop instrument.

Together, these reference systems reinforce the conclusions drawn from the cocoa butter measurements. When analysis is confined to a rigorously defined *q*-window and instrumental resolution effects are treated consistently, benchtop USAXS reproduces established scattering regimes across a range of fat-based soft-matter systems. At the same time, the results emphasize that interpretation must remain bound by instrumental constraints, particularly when complex multiphase materials are examined.

## 4 Conclusions

This study demonstrates how a benchtop ultra-small-angle X-ray scattering (USAXS) instrument can be used responsibly and reproducibly to probe micron-scale structural features in fat-based soft-matter systems, provided that instrumental limitations are explicitly defined and respected. Rather than pursuing comprehensive crystallization or polymorphism analysis, the focus has been on establishing practical best practices for data acquisition, treatment, and interpretation under laboratory conditions.

By using analyzer crystal rocking curves to define a lower bound for quantitative analysis and counting-statistics considerations to establish an upper bound, a reliable *q*-window was identified for the benchtop USAXS instrument examined here. Data outside this window were shown to be strongly influenced by instrumental effects, including direct-beam contributions and background variability, and were therefore excluded from interpretation. Within the validated range, slit-smearing effects inherent to Bonse–Hart geometries were treated through model convolution using widely available open-source software, enabling consistent extraction of power-law scattering regimes.

When applied to cocoa butter, commercial chocolate, and a reference triglyceride mixture, benchtop USAXS captured reproducible scattering trends within the accessible length scales. Comparisons with synchrotron SAXS measurements over overlapping *q*-ranges showed qualitative consistency of dominant scattering regimes, while also highlighting expected differences arising from sample variability and instrumental configuration. These results underscore that meaningful comparison across instruments requires restrained interpretation and explicit consideration of analysis boundaries.

Overall, this work provides a practical framework for the routine use of benchtop USAXS in soft-matter and food-materials research. By emphasizing interpretive limits alongside capabilities, it offers guidance for extracting reliable structural information from laboratory-scale measurements and supports the broader adoption of USAXS techniques beyond large-scale facilities.

## Author contributions

K. Q. K. T contributed to the planning and execution of all experimental procedures, data collection, analysis and the writing of the manuscript. A. G. M contributed to the experimental planning, carried out synchrotron SAXS experiments and contributed to the writing of the manuscript.

## Conflicts of interest

The authors declare no competing financial interest.

## Supplementary Material

RA-016-D6RA00082G-s001

## Data Availability

All supporting data can be accessed *via* the following link https://dataverse.harvard.edu/dataset.xhtml?persistentId=doi:10.7910/DVN/TRPOXG. Supplementary information (SI): scattering intensity differences of empty capillaries with different inner diameters. See DOI: https://doi.org/10.1039/d6ra00082g.
