# Supplementary material for: Multi-scale triglyceride crystal network analysis using a benchtop ultra-small-angle X-ray scattering instrument
Source: RSC Adv. 2026 Mar 5;16(14):12502–10. doi: 10.1039/d6ra00082g (PMC12961578; doi:10.1039/d6ra00082g)
Supplement: RA-016-D6RA00082G-s001 [file RA-016-D6RA00082G-s001.pdf]

Supplementary Information (SI) for RSC Advances.
This journal is © The Royal Society of Chemistry 2026

Multi-scale triglyceride crystal network analysis using a benchtop ultra-small-angle X-ray scattering instrument

*Kenneth Q. K. Truong, Alejandro G. Marangoni**

Department of Food Science, University of Guelph, Guelph, Ontario N1G2W1, Canada

*corresponding author, amarango@uoguelph.ca

Supplementary Information (3 pages)

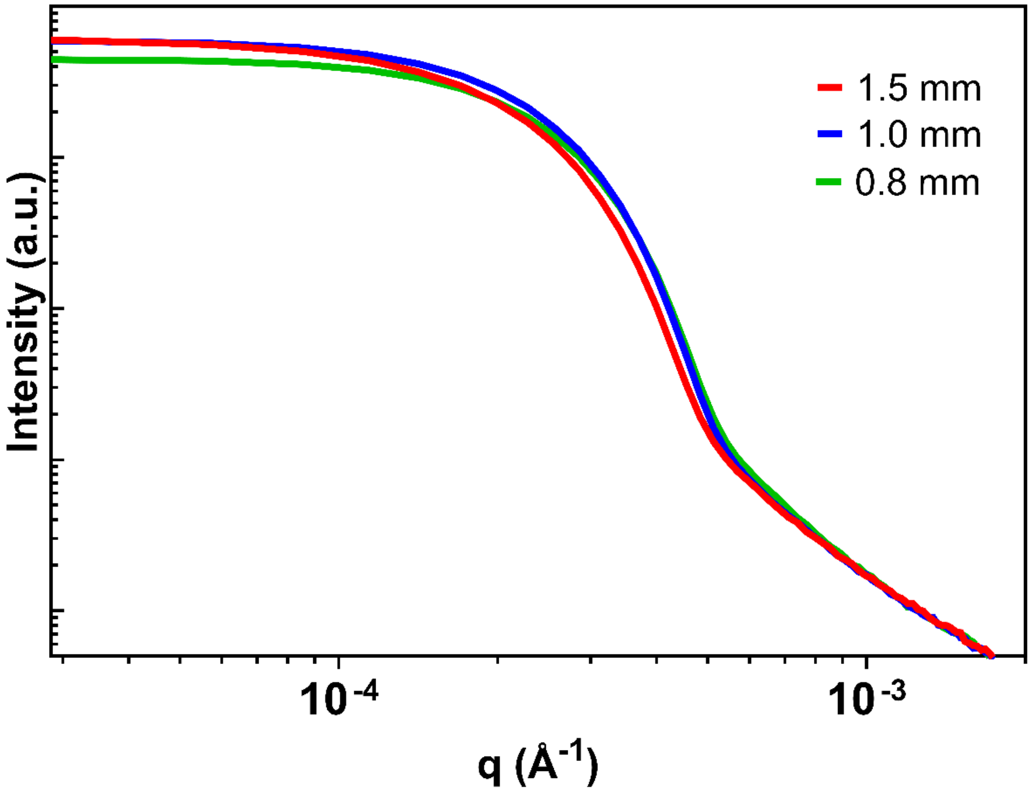

Supplementary Figure S1. Scattering intensity differences of empty capillaries with different inner diameters.

Supplementary Discussion

Effect of capillary diameter on blank scattering: Empty capillaries with inner diameters of 0.8mm, 1.0mm, and 1.5mm were measured on the Rigaku NANOPIX mini under identical conditions to assess how capillary size influences the background. Fig. 10 shows the difference in scattering intensities at very low q ($q < q_{min}$). Above q_{min}, the differences decrease and the curves approach a common baseline. Even for empty capillaries, intrinsic variations in the capillary itself can change the measured scattering baseline, necessitating replicate blank measurements.

Desmearing methods for slit-smeared data

Bonse-Hart USAXS instruments produce intrinsically slit-smeared 1D intensity profiles, such that the measured curve can be represented as the underlying scattering convolved with an effective 1D slit/beam profile. Several numerical desmearing routines have been developed for slit-collimated X-ray SAXS/USAXS data. Vad and Sager provide a detailed and practical discussion of iterative desmearing procedures for slit-smeared small-angle scattering data, including explicit convergence behavior and noise-handling considerations that are directly relevant when deciding between numerical desmearing versus model smearing during model fitting.[1] Two representative examples from their work are the Lake algorithm and the Van Cittert family of routines.[2,3] For the Lake approach, the iteration must be regulated by an objective stopping criterion because the solution reaches an optimum after a finite number of cycles and then deteriorates. Beyond the optimum, the data becomes overcorrected, and the artificial features can emerge. A key practical disadvantage is that the Lake desmearing can amplify statistical noise, requiring subsequent smoothing or noise filtering to recover a physically interpretable curve while preserving features. The Van Cittert routines can display the opposite trade-off. For noisy data, the procedure may become less strongly desmeared to maintain smoothness, and the number of iterations required for convergence can become large for high-throughput processing. Taken together, these documented limitations motivate the approach in this study. Rather than performing iterative deconvolution, which is sensitive to convergence choices and can either amplify noise or require extensive iteration, our objective was to present a faster, approachable method for handling slit-smeared benchtop USAXS data by model smearing.

1. T. Vad, W. F. C. Sager, Comparison of iterative desmearing procedures for one-dimensional small-angle scattering data, *Journal of Applied Crystallography*, 2011, **44**, 32–42.

2. J. Lake, An iterative method of slit-correcting small angle X-ray data, *Acta Crystallographica*, 1967, **23**, 191–194.

3. P.H. Van Cittert, *Z. Phys.,* 1931, **69**, 298–308.
